# Bamboo Kraft Pulp Black Liquor as a Renewable Source of Value-Added Carbon Dots

**DOI:** 10.3390/nano14231887

**Published:** 2024-11-23

**Authors:** Xiaolong Qiao, Shixing Wang, Qiulian Liu, Yuanming Zhang, Guangting Han, Haoxi Ben, Wei Jiang, Haiguang Zhao, Yan Song

**Affiliations:** 1 College of Textile and Clothing, Dezhou University, Dezhou 253026, China; qiaoxiaolong@qdu.edu.cn (X.Q.); kychgt@qdu.edu.cn (G.H.); 2 State Key Laboratory of Bio-Fibers and Eco-Textiles, College of Textiles and Clothing, Qingdao University, Qingdao 266071, China; wangshixing@qdu.edu.cn (S.W.); liuqiulian@qdu.edu.cn (Q.L.); zhangyuanming@qdu.edu.cn (Y.Z.); benhaoxi@qdu.edu.cn (H.B.)

**Keywords:** bamboo, black liquor (BL), carbohydrate, carbon dots (C-dots)

## Abstract

China is the country with the most abundant bamboo resources in the world. Using bamboo as a raw material for pulping and papermaking can save a lot of wood and protect forests. Bamboo pulping enterprises mostly adopt sulfate processes to produce a large amount of black liquor (BL), which contains monosaccharides, polysaccharides, oligosaccharides, pectin, lignin, etc. The utilization of the high-value organic matter is of great economic and environmental significance. In this study, blue-green carbon dots (C-dots) were prepared from bamboo (*Lingnania chungii*) kraft pulp BL using a hydrothermal method. The changes in carbohydrate content in BL in relation to hydrothermal temperature and hydrothermal time were discussed in detail. Then, a series of characterizations of BL-C-dots, prepared under one of the hydrothermal conditions (180 °C, 6 h), were performed and the BL-C-dots showed an excitation-dependent photoluminescence (PL) spectrum and a quantum yield (QY) of 2.9% in an aqueous solution. Finally, the as-prepared BL-C-dots were successfully used as fluorescent materials to develop an anti-counterfeiting code. The fluorescent code exhibited a clear outline, an excitation-tunable color, good stability, and high security, showing great anti-counterfeiting potential and realizing the high-value utilization of BL.

## 1. Introduction

China is the country with the most abundant bamboo resources in the world, with more than 500 varieties of bamboo [1], and its planting area and storage volume rank first in the world [2]. Due to the shortage of raw materials in China’s pulp and paper industry, bamboo is an important raw material supplement [3]. Using bamboo as a raw material for pulping and papermaking can save a lot of wood and protect forests [4].

Bamboo pulping enterprises mostly adopt sulfate processes, which produce a large amount of wastewater [5,6,7]. Bamboo kraft pulp black liquor (BL) accounts for 88% of the total pollutants in bamboo kraft pulp wastewater [3]. The uncontrolled discharge of BL can cause serious pollution in the environment [8]. At present, most enterprises use alkali recovery technology to treat BL [9,10]. BL is usually sent to alkali recovery furnaces after evaporation and concentration to generate heat and energy [11,12], along with large amounts of CO_2_ and SO_2_, resulting in air pollution [13]. However, the potential value of BL is ignored. BL contains a lot of organic matter, such as cellulose, hemicellulose, lignin, monosaccharides, polysaccharides, oligosaccharides, pectin, etc. [14]. Converting these components into high-value by-products can not only reduce the pollution of the environment with wastewater, but can also yield high-cost products [15]. BL is a prospective lignocellulosic feedstock for the development of practices in line with a circular bioeconomy [16].

In recent years, the bioconversion of BL compositions into bioplastics, biohydrogens, biogases, and chemicals has shown good progress [14,17], but there are few reports on the preparation of carbon dots (C-dots) from BL compositions [18]. C-dots prepared from biomasses have become promising zero-dimensional carbon nanomaterials [19]. They not only have the advantages of traditional semiconductor quantum dots, such as strong luminescence, tunable excitation/emission, and excellent electron transfer ability [20], but also avoid the shortcomings of traditional semiconductor quantum dots, such as poor biocompatibility and high toxicity [21]. Additionally, they have potential application prospects in biology, medicine, printing, chemistry, energy, and other fields [22]. Biopolysaccharides present in BL can be used to prepare C-dots [23].

C-dots can be synthesized using top-down and bottom-up approaches [24]. The “top-down” approach includes laser ablation and electrochemical approaches [25], whereas the “bottom-up” approach includes thermal pyrolysis, hydrothermal, chemical oxidation, and microwave methods [26]. The top-down method refers to the physical, chemical, or electrochemical breakdown of larger particles into smaller ones on the nanometric scale [27]. However, in the bottom-up approach, small molecules are pyrolyzed or carbonized to form nano-particles with the desired size range [28].

In this study, BL-C-dots were prepared from bamboo (*Lingnania chungii*) kraft pulp BL using a hydrothermal method. The changes in the carbohydrate content in BL with hydrothermal temperature and hydrothermal time were discussed in detail. Then, we prepared a series of characterizations of BL-C-dots, prepared under one of the hydrothermal conditions, including assessments of morphology, size, surface chemical composition, fluorescence decay time, quantum yield (QY), etc. Finally, the as-prepared BL-C-dots were used as fluorescent materials for the development of anti-counterfeiting code. This approach showed a high level of security and realized the high-value utilization of BL.

## 2. Experimental

### 2.1. Materials

BL (Na_2_SO_3_ cooking process) was obtained from the Institute of Chemical Industry of Forest Products, Chinese Academy of Forestry, Nanjing, China. Sulfuric acid (H_2_SO_4_) was purchased from Sinopharm Chemical Reagent Co., Ltd., Shanghai, China. Glucose, xylose, and arabinose were obtained from Aladdin Reagent Co., Ltd., Shanghai, China, and were purified by chromatography.

### 2.2. Analysis of the BL

The monosaccharide content test was performed as follows: The content of monosaccharides in BL was determined via HPLC. We adjusted the pH of BL to 2. After centrifugation (15,000 r/min, 20 min), the supernatant was filtered and tested. The HPLC conditions were as follows: the chromatography was performed on the Bio. Rad Aminex HPX-87H column, with a 0.002 mol/L H_2_SO_4_ eluent, at a flow rate of 0.6 mL/min, a column temperature of 50 °C, a sample size of 10 μL, and a differential refraction detector (RID, 45 °C).

The polysaccharide content test was performed as follows: BL (50 mL, treated for lignin precipitation) was mixed with H_2_SO_4_ (72 wt%) until the pH reached 1.7 (4 wt% H_2_SO_4_). This was followed by hydrolysis at 121 °C for 1 h. The hydrolyzed wastewater was then centrifuged, and the supernatant was filtered using a filter film with a pore size of 0.22 μm. This filtrate was analyzed by HPLC under the same conditions as outlined above. The polysaccharide content in BL was calculated based on the monosaccharide content obtained from hydrolysis. The glucose content measured was multiplied by a coefficient of 0.9 to determine the glucan content, while the xylose and arabinose contents were multiplied by a coefficient of 0.88 to obtain the respective xylan and araban contents.

### 2.3. Preparation of BL-C-Dots

**Figure 1:**
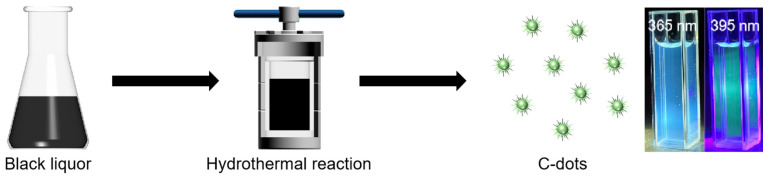
The preparation process of BL-C-dots.

The BL (10 mL of the supernatant after centrifugation) was transferred to a hydrothermal reactor and subjected to a reaction in an oven (temperature of 180 °C; time gradient of 6 h, 8 h, 10 h, 12 h, and 14 h; time of 6 h; temperature gradient of 140 °C, 160 °C, 180 °C, and 200 °C). Subsequently, the BL was dialyzed for 48 h and freeze-dried. The resulting dried powder was collected for further characterization and the fabrication of security codes. The preparation process of the BL-C-dots is schematically shown in Figure 1.

### 2.4. Characterizations

The morphology and structure of the BL-C-dots were measured using a transmission electron microscope (TEM, JEOL 2100F, Tokyo, Japan); the X-ray diffraction (XRD, Rigaku MiniFlex 600, Tokyo, Japan) spectrum was recorded to analyze the crystal structure of BL-C-dots; an X-ray photoelectron spectrometer (XPS, ESCALAB Xi+, Thermo Fisher Scientific, Waltham, MA, USA) and a Fourier transform infrared spectrometer (FT-IR, Nicolet 6700, Hudson, WI, USA) were used to characterize the surface chemical structure of BL-C-dots; the absorption characteristics of the BL-C-dots were tested using a UV-visible spectrophotometer (UV-2600, Shimadzu, Kyoto, Japan); the fluorescence spectrum, fluorescence lifetime, and photoluminescence quantum yield (PLQY) of the BL-C-dots were characterized using a steady state/transient fluorescence spectrometer (FLS1000, Edinburgh Instruments, Edinburgh, UK); and the Zeta potential was measured using a Malvern Zetasizer Nano ZSE (DLS-Zeta potential, Malvern Panalytical, Malvern, UK).

### 2.5. Preparation of Fluorescent Ink and Patterns

The BL-C-dot solution was first filtered using a 0.45 μm filter, followed by a 0.22 μm filter, to produce fluorescent ink. This ink was then printed on non-fluorescent paper using an ink-jet printer (Canon, Tokyo, Japan, HFTX-P4290C).

## 3. Results and Discussion

### 3.1. Changes in Carbohydrate Content in BL Under Varying Hydrothermal Conditions During C-Dot Preparation

In this study, we conducted a comprehensive analysis of the chemical composition of BL to identify which component was critical for C-dot synthesis. Table 1 and Table 2 explore the changes in carbohydrate content in BL under varying hydrothermal conditions, providing essential insights into the influence of reaction time and temperature on the formation of C-dots.

Table 1 presents the changes in monosaccharide and polysaccharide content in BL as a function of hydrothermal reaction time, with samples named according to their reaction time (e.g., BL-6 h represented a sample treated for 6 h). The results indicated a significant decrease in the concentration of monosaccharides (glucose, xylose, and arabinose) and polysaccharides (glucan, xylan, and araban) as the reaction time increased at a constant temperature of 180 °C. For example, the glucose concentration decreased markedly from 2.55 ± 0.05 g/L in untreated BL to 0.57 ± 0.12 g/L after a 6 h reaction, highlighting its rapid conversion into carbon-rich structures under hydrothermal conditions. This suggested that monosaccharides undergo dehydration and carbonization, serving as the primary carbon sources for C-dot formation.

The decrease in polysaccharides was less pronounced compared to that seen in monosaccharides, indicating a sequential conversion process. Polysaccharides like glucan and xylan were likely hydrolyzed into monosaccharides before they contributed to carbonization. The residual content of polysaccharides after an extended reaction time suggested that these larger macromolecules degraded more slowly, thus serving as secondary sources of carbon. This analysis confirmed that monosaccharides, especially glucose, played a pivotal role in the initial stages of C-dot synthesis, while polysaccharides extended the carbon availability over prolonged hydrothermal treatment.

Table 2 demonstrates the changes in the carbohydrate composition of BL under varying hydrothermal temperatures, with samples such as that named BL-140 °C denoting the reaction temperature, while a reaction time of 6 h is maintained. At lower temperatures (140 °C and 160 °C), the residual monosaccharide content remained higher, indicating incomplete conversion. As the temperature increased to 180 °C, over 93% of the monosaccharides were converted, achieving a high yield of C-dots. This temperature appeared optimal for facilitating the dehydration and polymerization processes that transformed these carbohydrates into carbon structures.

At 200 °C, while further reductions in carbohydrate content were observed, the degradation of monosaccharides was accompanied by an increase in side reactions and by-products, potentially leading to over-carbonization. This condition might produce more amorphous or irregular carbon structures, potentially compromising the uniformity and PL properties of the C-dots. Therefore, 180 °C was identified as the ideal temperature, balancing efficient conversion with the production of high-quality C-dots. These observations showed that the reaction temperature not only accelerated the conversion rate but also determined the quality of the C-dots by influencing the balance between carbonization and the formation of by-products.

The data from the tables emphasize that glucose and other monosaccharides acted as primary precursors, while polysaccharides contributed progressively through hydrolysis and carbonization, supporting the continuous synthesis process. Optimizing these parameters could enable the more effective utilization of biomass to produce high-value carbon nanomaterials.

In order to further analyze the structures and properties of C-dots, we selected BL-C-dots, prepared with a hydrothermal reaction time of 6 h and hydrothermal reaction temperature of 180 °C, as the characterization objects, and applied them in anti-counterfeiting printing. Detailed data were provided in the following sections.

### 3.2. Morphology and Structure of BL-C-Dots

**Figure 2:**
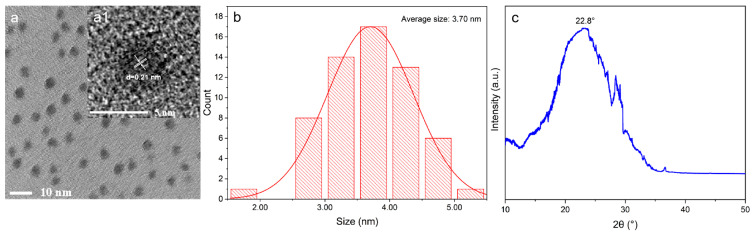
(**a**) A TEM image of BL-C-dots. (**a1**) An HRTEM image of the BL-C-dots. (**b**) The size distribution of the BL-C-dots. (**c**) The XRD pattern of the BL-C-dots.

The morphology and size of the as-prepared BL-C-dots were characterized by transmission electron microscopy (TEM). As shown in Figure 2a,b, the BL-C-dots typically exhibited spherical shapes with an average particle size of 3.70 nm. The observed d-spacing was approximately 0.21 nm (Figure 2(a1)), which closely corresponded to that of graphite carbon [29]. The clear lattice showed that the BL-C-dots had a high crystalline structure due to high-temperature carbonization [30]. The X-ray diffraction (XRD) pattern exhibited a broad peak at ≈22.8° (Figure 2c), corresponding to the (002) planes of graphitic carbon, suggesting the *sp*^2^ hybrid carbon–core structure of BL-C-dots [31].

**Figure 3:**
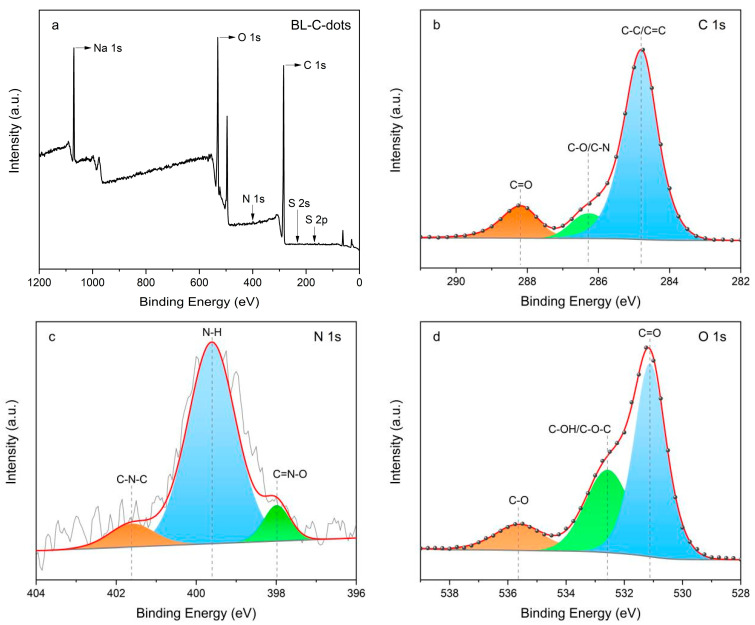
(**a**) The full-survey XPS spectrum of the BL-C-dots. (**b**–**d**) The high-resolution XPS spectra of the C l s, N 1s, and O 1s of the BL-C-dots.

**Figure 4:**
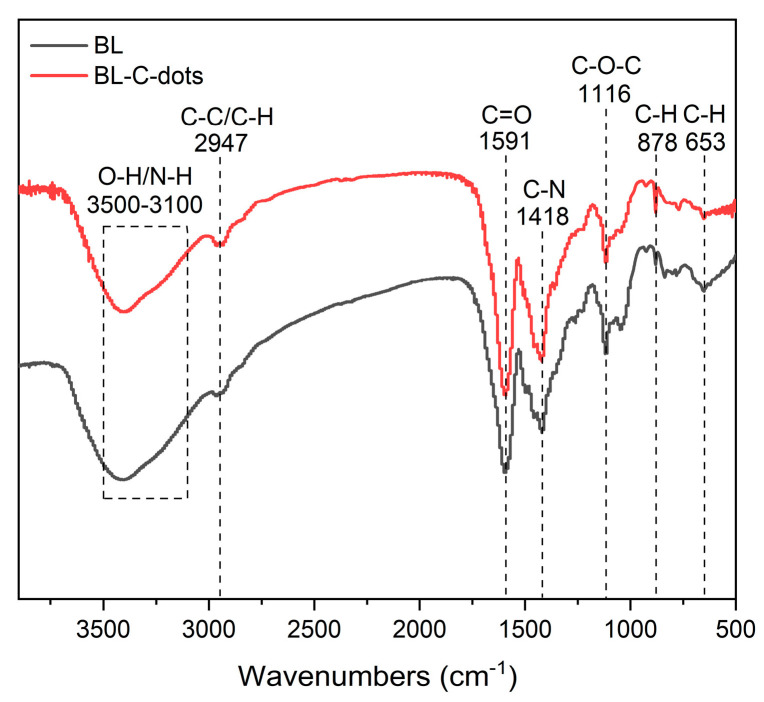
FT-IR spectra of BL and BL-C-dots.

The surface chemical composition of the BL-C-dots was analyzed using XPS, and the results are shown in Table 3 and Figure 3. Based on the full survey of XPS (Figure 3a), it can be stated that the as-prepared BL-C-dots consisted of C, N, and O elements, with atomic percentages of 57.03%, 1.28%, and 29.33%, respectively. The presence of Na and S might correspond to the presence of Na_2_SO_3_ in BL. The spectrum of the C 1s could be divided into three bonds, located at 284.8 eV, 286.3 eV, and 288.2 eV. These were assigned to C–C/C=C, C–O/C–N, and C=O bonds [18], respectively (Figure 3b). The spectrum of the N 1s could be divided into three bonds located at 397.9 eV, 399.6 eV, and 401.6 eV, which were assigned to C=N–O, N–H, and C–N–C bonds [32], respectively (Figure 3c). Figure 3d showed the O 1s of the BL-C-dots, with peaks of 531.1 eV, 532.6 eV, and 535.6 eV, could be attributed to C=O, C–OH/C–O–C, and C–O bonds [33], respectively. The FT-IR spectra (Figure 4) reveal a range of characteristic absorption bands that correspond to various functional groups, including O–H/N–H, C–C/C–H, and C=O bonds. The presence of O-H/N-H stretching vibrations indicated that the C-dots possessed hydroxyl and amine functional groups, which could enhance their solubility in aqueous solutions and improve their stability in various environments [15,34]. The C=O stretching observed in the FT-IR spectra suggested the presence of carbonyl groups, which could play a role in the PL properties of the BL-C-dots by facilitating energy transfer mechanisms [23,35]. The detection of these functional groups aligned with the XPS analysis, reinforcing the conclusion that the surface chemistry of the BL-C-dots was rich and varied. In addition, the functional groups of BL and BL-C-dots in Figure 4 were the same, and no new functional groups appeared, indicating that BL was the precursor of BL-C-dots.

### 3.3. Optical Properties of BL-C-Dots

**Figure 5:**
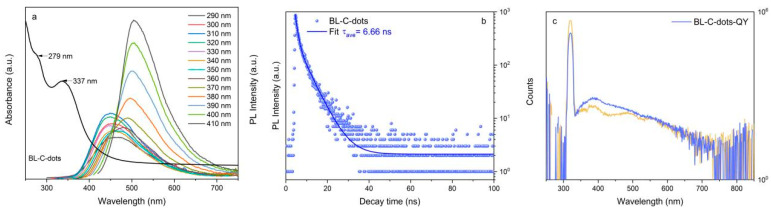
(**a**) UV absorption spectra of BL-C-dots and PL spectra of BL-C-dots excited at different wavelengths. (**b**) The fluorescence decay curve of the BL-C-dots. (**c**) The QY of the BL-C-dots.

The as-prepared BL-C-dots had a typical absorption below 500 nm, which was similar to the value seen for C-dots prepared with glucose or xylose [15]. The BL-C-dots showed excitation-dependent fluorescence spectra with excitation wavelengths ranging from 290 nm to 410 nm (Figure 5a). These values were also similar to those of the C-dots produced by glucose or xylose [15]. This was because the wastewater also contained glucose or xylose, resulting in the same C-dot structure. The excitation-dependent photoluminescence (PL) behavior in C-dots typically arose from a low crystalline core structure and loosely packed surface functional groups, resulting in excitation-dependent energy states [36]. In this work, the behavior was primarily attributed to the loosely packed surface functional groups, as the highly crystalline core structure of the BL-C-dots was enhanced by the high-temperature hydrothermal reaction. As shown in Table 4, the BL-C-dots exhibited an average fluorescence lifetime of 4.09 ns, which was characteristic of C-dots and suggested efficient fluorescence decay. This relatively short lifetime, further demonstrated in the fluorescence decay curve in Figure 5b, indicated that the BL-C-dots could rapidly release energy. In addition, Figure 5c showed that the QY of the BL-C-dots was measured at 2.9%. Although this QY was moderate compared to the values of some higher-efficiency nanomaterials [32,35], it was comparable to the values of other biomass-derived C-dots, indicating good PL properties [15,18,23]. The excitation-dependent fluorescence and stable optical performance of the BL-C-dots make them promising candidates for applications such as anti-counterfeiting, where tunable fluorescence can enhance security features.

### 3.4. Printing of Anti-Counterfeiting Pattern

**Figure 6:**
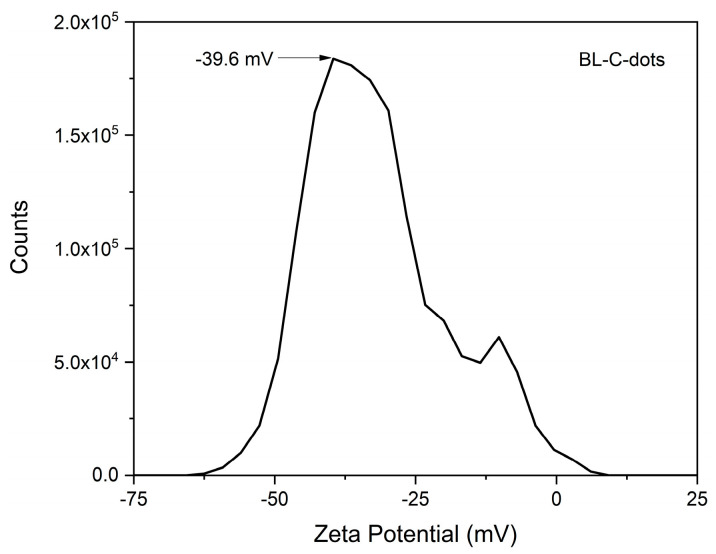
Zeta potential curve of the BL-C-dots.

The Zeta potential curve showed that the surface of BL-C-dots was negatively charged and exhibited good stability in an aqueous solution, meaning that BL-C-dots had excellent water solubility [37] (Figure 6).

**Figure 7:**
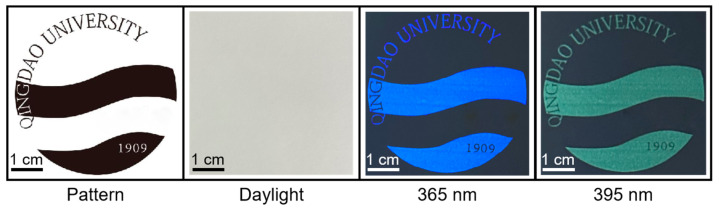
The designed and printed fluorescent pattern on non-fluorescent paper with BL-C-dot ink under room light and illumination conditions featuring 365 and 395 nm lights.

A fluorescent pattern was printed on non-fluorescent paper using BL-C-dots ink. As shown in Figure 7, under indoor lighting, there were no visible patterns on the paper, but under illumination with 365 nm and 395 nm lights, clear fluorescent patterns could be seen. The pattern appeared blue under 365 nm illumination and green under 395 nm illumination due to the excitation-dependent PL behavior of the BL-C-dots. Moreover, BL-C-dots did not affect the flexibility of the paper. This result indicated that we could develop a flexible fluorescent code for multi-level anti-counterfeiting.

## 4. Conclusions

In this work, we successfully prepared blue-green C-dots from BL using a hydrothermal method. A comprehensive analysis of the carbohydrate composition of the BL revealed how variations in hydrothermal reaction temperature and time influenced the formation of BL-C-dots. The results showed that monosaccharides, particularly glucose, played a critical role in the carbonization process, while polysaccharides contributed progressively during hydrolysis. The BL-C-dots exhibited excellent properties, including an excitation-dependent PL spectrum, a QY of 2.9%, and a stable crystalline structure, making them highly promising for practical applications. Finally, the as-prepared BL-C-dots were successfully applied in anti-counterfeiting printing, demonstrating high security, good stability, and tunable fluorescence colors under different excitation wavelengths. This work not only provides a new approach for the high-value utilization of BL, but also highlights the potential of biomass-based C-dots in advanced material applications such as anti-counterfeiting technology. These findings pave the way for the further exploration of renewable resources in the development of functional nanomaterials, contributing to both environmental sustainability and a circular bioeconomy. 

## Figures and Tables

**Table 1:** Changes in monosaccharide and polysaccharide content in BL with hydrothermal reaction time at 180 °C.

Sample	Monosaccharide (g/L)			Polysaccharide (g/L)		
	Glucose	Xylose	Arabinose	Glucan	Xylan	Araban
BL	2.55 ± 0.05	1.05 ± 0.00	1.03 ± 0.17	2.82 ± 0.17	1.95 ± 0.02	0.81 ± 0.01
BL-6 h	0.57 ± 0.12	0.72 ± 0.01	0.76 ± 0.06	1.92 ± 0.06	0.13 ± 0.00	0.17 ± 0.03
BL-8 h	0.49 ± 0.01	0.70 ± 0.03	0.77 ± 0.12	1.52 ± 0.12	0.07 ± 0.03	0.16 ± 0.03
BL-10 h	0.48 ± 0.07	0.66 ± 0.09	0.75 ± 0.11	1.20 ±0.11	0.06 ± 0.11	0.12 ± 0.01
BL-12 h	0.42 ± 0.07	0.63 ± 0.05	0.68 ± 0.03	1.15 ± 0.03	0.05 ± 0.02	0.10 ± 0.05
BL-14 h	0.40 ± 0.02	0.64 ± 0.06	0.63 ± 0.00	1.10 ± 0.00	0.01 ± 0.01	0.04 ± 0.11

**Table 2:** Changes in the monosaccharide and polysaccharide content in BL with the hydrothermal reaction temperature under the condition of a hydrothermal reaction time of 6 h.

Sample	Monosaccharide (g/L)			Polysaccharide (g/L)		
	Glucose	Xylose	Arabinose	Glucan	Xylan	Araban
BL	2.55 ± 0.05	1.05 ± 0.00	1.03 ± 0.17	2.82 ± 0.17	1.95 ± 0.02	0.81 ± 0.01
BL-140 °C	2.50 ± 0.00	0.83 ± 0.06	0.84 ± 0.03	2.82 ± 0.03	1.90 ± 0.06	0.74 ± 0.03
BL-160 °C	2.49 ± 0.10	0.74 ± 0.00	0.79 ± 0.01	2.07 ± 0.01	1.89 ± 0.01	0.60 ± 0.12
BL-180 °C	0.57 ± 0.12	0.72 ± 0.01	0.76 ± 0.06	1.92 ± 0.06	0.13 ± 0.00	0.17 ± 0.03
BL-200 °C	0.45 ± 0.02	0.64 ± 0.03	0.09 ± 0.01	1.02 ± 0.01	0.04 ± 0.01	0.14 ± 0.07

**Table 3:** Atomic percentages of the BL-C-dots elements.

Elements	C	N	O	Na	S
Atomic percent (%)	57.03	1.28	29.33	10.05	2.31

**Table 4:** The decay time and QY of the BL-C-dots.

Sample	T1 (ns)	T2 (ns)	Average Lifetime (ns)	QY (%)
BL-C-dots	1.10	5.56	4.09	2.9

## Data Availability

Data are contained within the article.
